# Impact of factor rotation on Q-methodology analysis

**DOI:** 10.1371/journal.pone.0290728

**Published:** 2023-09-01

**Authors:** Noori Akhtar-Danesh

**Affiliations:** 1 School of Nursing, McMaster University, Hamilton, Canada; 2 Department of Health Research Methods, Evidence and Impact, McMaster University, Hamilton, Canada; Max Planck Institute for Solid State Research, GERMANY

## Abstract

The Varimax and manual rotations are commonly used for factor rotation in Q-methodology; however, their effects on the results may not be well known. In this article we investigate the impact of different factor rotation techniques in Q-methodology, specifically how the factors and their distinguishing statements might be affected. We applied three factor rotation techniques including Varimax, Equamax, and Quartimax rotations on two exemplary datasets and compared the results based on the number of Q-sorts loaded on each factor, number of distinguishing statements for each factor, and changes in the number of distinguishing statements. We also estimated the Pearson correlation between the extracted factors based on rotation techniques. This analysis shows that factors can change substantially from one rotation to another. For instance, there was only 3 common distinguishing statements between Factor 1 of no-rotation of Dataset 1 and its matched factor from Varimax rotation. Even for 3 common statements, the factor scores were quite different from no-rotation to Varimax rotation. This analysis shows that the effects of factor rotation on emerging factors are complex. The changes are usually substantial such that the rotated factors might be quite different from the original factors.

## Introduction

Q-methodology is a research method which is a combination of quantitative and qualitative techniques. The statistical component of Q-methodology includes factor extraction and factor rotation. The most common factor extraction methods in Q-methodology are principal component and centroid factor extraction [1–4], while the most common techniques for factor rotation are Varimax rotation and manual rotation [1–4]. These factor extraction and factor rotation techniques are the only methods available in the widely used Q-specific programs such as PQMethod [5] and KADE [6]; however, more factor extraction methods such as principal axis factoring and factor rotation methods such as Quartimax and Equamax are available in the newly developed programs Q FACTOR [7] and QMETHOD [8].

Although the abovementioned factor rotation techniques are commonly used in Q-methodology analysis, their effects on the results may not be well-known. For example, a recent book chapter from a renowned Q-methodologist claims that there is not much difference in the results of Q-methodology analysis based on different factor rotation techniques [9].

In this article we aimed to study the impacts of different rotation techniques on the results of Q-methodology analysis, specifically examining how the factors and their distinguishing statements might change based on factor rotation. We introduce two exemplary datasets and apply different factor rotation techniques and compare different indices based on factor rotation to show the extent of change in the rotated factors. These indices include number of Q-sorts loaded on each factor, number of distinguishing statements, changes in the distinguishing statements, changes in the number of distinguishing statements, and correlation between factors scores based on different rotations.

## Methods & material

### Q-methodology

**Fig 1:**
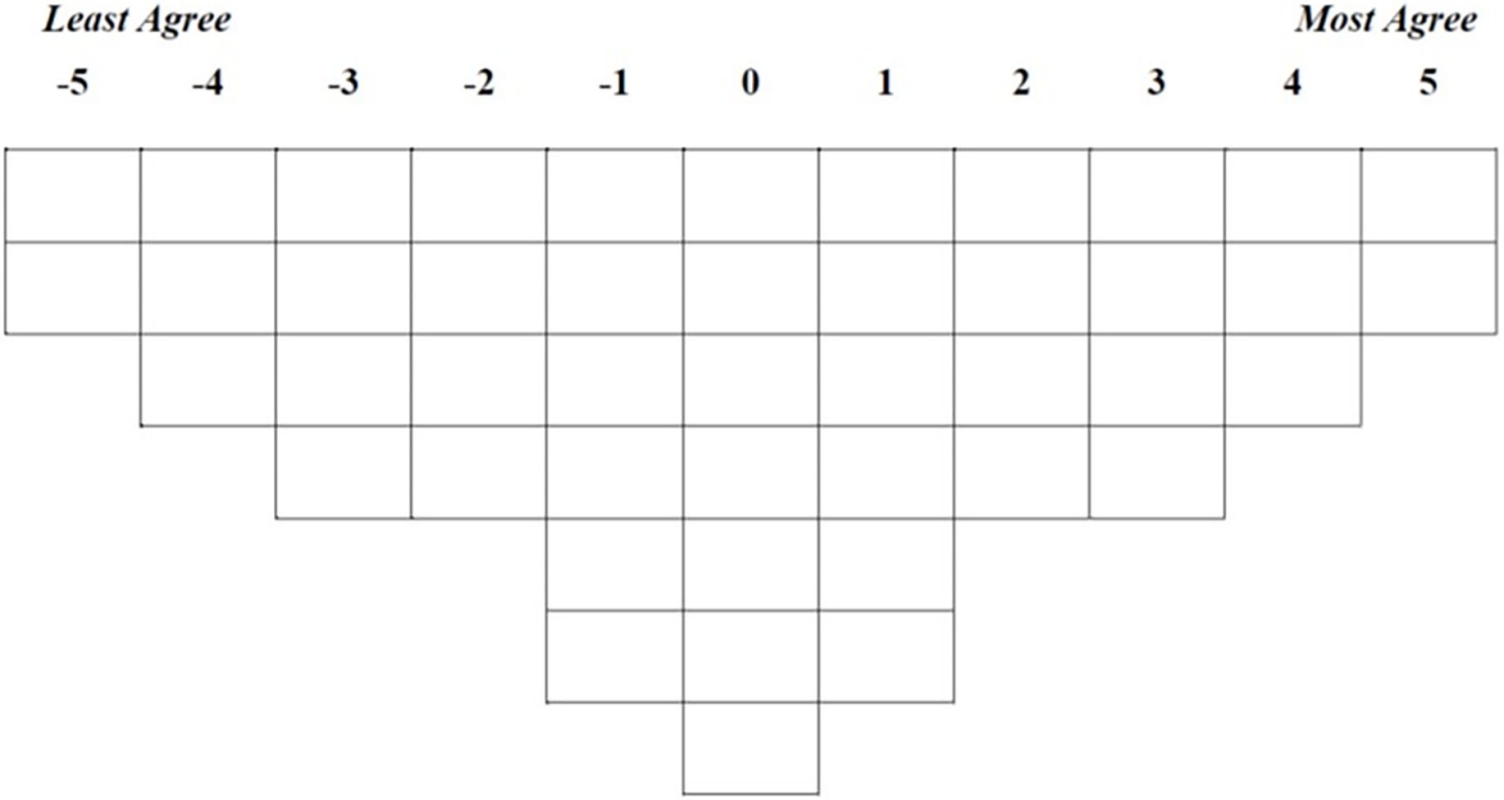
A Q-sort table with anchors of -5 and +5.

Q-methodology was introduced by Stephenson in 1935 [10,11] as a research methodology and is used to identify shared and salient viewpoints among study participants. A Q-methodological study involves several steps including: a) development of a sample of statements related to the topic of interest known as Q-sample or Q-set, b) rank-ordering of the Q-sample by the participants based on individual views and preferences using a grid (known as Q-sort table) with a quasi-normal distribution (e.g. see Fig 1), c) a by-person factor analysis (i.e., the factor analysis is performed on persons, not variables or traits) to analyze the completed Q-sort tables known as Q-sorts, where each Q-sort represents one individual rather than a variable or trait, and d) interpretation of the results [12–15]. For the rest of this article, we use the terms Q-sort and variable interchangeably. S1 Appendix includes a glossary of key terms used in Q-methodology.

In the analysis stage, using a by-person factor analysis, similar Q-sorts (individuals) are grouped together as factors, where each factor represents a group of individuals with similar views, feelings, or preferences about the theme of the study. Then, each factor is interpreted based on its *distinguishing statements* and statements with high or low factor scores. A distinguishing statement is a statement for which the factor score is significantly different from factor scores of other factors. Distinguishing statements usually define the uniqueness of each factor.

### Factor extraction methods

The general statistical programs include several factor extraction methods such as principal component analysis (PCA), principal axis factoring (PAF), maximum likelihood (ML) factoring, image factoring, and alpha factoring. However, for a study with a large sample, the differences in the extracted factors are usually negligible [16]. For a detailed review of factor extraction methods in Q-methodology, the reader is referred to Akhtar-Danesh [1,17].

### Factor rotation techniques

A common phenomenon in factor analysis is that the original extracted factors may not be meaningful or easily interpretable, regardless of what factor extraction technique is used. As a result, the factors need to be rotated to have a simpler structure and to be more interpretable [18]. Therefore, the original factors are rotated about their origin to achieve this goal. The most common approaches to factor rotation in Q-methodology are Varimax rotation and manual rotation [1,2], although recently some other rotation techniques have became available to Q-methodologists. For instance, both QMETHOD [8] and QFACTOR [7] programs provide a range of other techniques such as Quartimax and Equamax for orthogonal rotation and direct oblimin for oblique rotation.

In this section we review five common factor rotation techniques including Varimax, Quartimax, Equamax, Direct oblimin, and Promax. The manual rotation which is occasionally used in Q-methodology [2], is not described for two reasons; first we intend to compare some more objective rotation solutions and manual rotation is rather subjective. Second, because of its subjective nature manual rotation might be less comparable with other objective approaches [1,16,19].

*Varimax* is the most common rotation technique in statistical analysis. To simplify the factor interpretation, this orthogonal rotation minimizes the number of variables with high loadings for each factor. Indeed, for each factor it maximizes the variance of its loadings by making high loadings higher and low loadings lower. In Q-methodology analysis, Varimax rotation redistributes the total variance among Q-sorts between a smaller number of factors with relatively equal variances [16]. From a mathematical point of view, this process does not allow a “general” factor to be emerged even if one such factor exists among the Q-sorts [20,21]. Therefore, using a Varimax rotation may not be suitable if a “general” factor exists among the Q-sorts.

*Quartimax* is an orthogonal method to minimize the number of factors that explain each Q-sort so that each Q-sort is loaded on the minimum number of factors. It simplifies the rows of the factor-loading matrix by loading each Q-sort strongly on a single factor. To some extent, this is a desired process in Q-methodology where each Q-sort is preferred to be loaded only on one factor. This process provides a more interpretable factor compared to varimax rotation. This method tends to generate a general factor among the participants [22]. A general factor usually consists of a large number of Q-sorts compared to the other factors. Therefore, if the existence of a general factor is expected among the Q-sorts, this method might be the method of choice; however, it may create a general factor even if one does not exist among the Q-sorts.

*Equamax* rotation is another orthogonal rotation technique. Mathematically, it is a combination of Varimax and Quartimax techniques that simplifies both the number of variables that load highly on a factor and the number of factors needed to explain a variable [16].

*Direct oblimin* is an oblique (non-orthogonal) rotation method. This technique minimizes the cross products of loadings to simplify factors. Direct oblimin allows the factor to be correlated, although factors may not necessarily correlate if this method is used. *Promax* is also another oblique rotation but might work faster than a direct oblimin rotation [16]. Therefore, it might be the preferred method for working with large datasets.

It is worth mentioning that these rotation methods preserve the same subspace but give a different basis for it. Also, the choice of rotation method depends on the research question and the underlying structure of the data. For instance, if the data is highly correlated, an oblique rotation may be appropriate, while a method like Varimax may be more robust if the underlying factors are relatively independent. On the other hand, if the researcher has a theory or hypothesis in mind for the factors’ positions in the data space, then use of a theoretical (or manual) rotation might be more appropriate [19,23]. In general, different rotation techniques tend to give similar results if the pattern of correlations in the dataset is fairly clear or if there is a stable solution for the dataset [16,21].

In the following, we first introduce two datasets, followed by a detailed plan for factor extraction and factor rotation.

### Dataset 1: Marijuana legalization

This dataset includes 40 participants from different Q-methodology workshops that the author has conducted in different countries (the dataset is provided in S1 File). The participants were asked to rank-order 19 statements (Q-sample) regarding their views on marijuana legalization. The Q-sort table included 5 rows and 7 columns with anchors of -3 (*Strongly Disagree*) and +3 (*Strongly Agree*) [24]. The list of statements is provided in S2 Appendix.

### Dataset 2: Childhood obesity

In this study parents’ perceptions on childhood obesity, its impact on children’s health, and the barriers in preventing childhood obesity were investigated. Participants included parents attending a well-baby check-up clinic. In total, 33 parents participated in the study which included a Q-sample with 42 statements (the dataset is provided in S2 File). The Q-sort table had 8 rows and 11 columns with anchors of -5 (*Strongly Disagree*) and +5 (*Strongly Agree*). The study is fully described by Akhtar-Danesh et al. [25].

### Plan for factor extraction and factor rotation

In the present study, we first used PCA and PAF for factor extraction. In general, PCA is the most common factor extraction method. It is also used more than any other method in Q-methodology [2]. Although, use of centroid factor analysis is quite common in Q-methodology [2], we used PAF instead because the centroid method is mathematically an approximation of PAF [26,27]. We extracted 3 factors based on each combination of factor extraction and factor rotation for Dataset 1. We similarly extracted 3 factors based on PCA for Dataset 2 and the abovementioned 3 factor rotations. We chose to extract 3 factors for two reasons; first, it is complex enough to show the changes that might happen in the factor indices investigated in this analysis. Second, more than 3 factors make the comparison between these indices much more complex. Next, we applied three factor rotation techniques including Varimax, Equamax, and Quartimax rotations to compare the results of factor rotation techniques with no-rotation and with each other. These rotation techniques were chosen as they are common techniques in the statistical literature [1,17]. We did not use a manual rotation or oblique rotation because use of the previously mentioned methods (Varimax, Equamax, and Quartimax rotations) is sufficient for the purpose of this article. Then, we compared the results based on the number of Q-sorts loaded on each factor, number of distinguishing statements on each factor, and changes in the number of distinguishing statements when compared with *no-rotation*. The distinguishing statements were identified using a Cohen’s effect size of 0.80 [28]. Next, we calculated the Pearson correlation coefficients between the extracted factors based on different rotation techniques. Finally, each rotated factor from Varimax, Equamax, and Quartimax rotations was matched with a factor from no-rotation based on the largest absolute value of correlation (marked with asterisk in Tables 2 and 3). The distinguishing statements are provided for Dataset 1 based on no-rotation and the other rotation techniques. The QFACTOR program was used for analysis [7] (see Table 4). S3 File includes the statistical codes.

This study involves secondary analysis of two anonymized datasets from two studies which had ethics approval from the Health Sciences Research Ethics Board, McMaster University. Participants consents were obtained verbally for Dataset 1 and written consent were obtained for Dataset 2.

## Results

Table 1 includes number of Q-sorts loaded on each factor and number of distinguishing statements based on factor extraction and factor rotation techniques for the two datasets. In general, there is not a large difference in the number of loaded Q-sorts based on factor extraction methods, but there seems to be substantial differences based on factor rotation techniques. The difference in the loaded Q-sorts is mostly between no-rotation and Quartimax rotation, compared to Varimax and Equamax rotations. For instance, the number of Q-sorts loaded on Factor 1 from no-rotation with PCA extraction on Dataset 1 changed from 22 to 13, 10, and 20 for Varimax, Equamax, and Quartimax rotations, respectively. Therefore, each rotated matched factor might represent a different set of Q-sorts, although there still might be a shared number of Q-sorts loaded on both factors. For example, on Factor 1 from no-rotation to Varimax rotation, where the number of Q-sorts changed from 22 to 13, the matched factor seems quite different as, at the very least, there are 9 fewer Q-sorts on the matched factor compared to the no-rotation factor.

**Table 1:** Factor characteristics based on different factor extraction and factor rotation techniques.

Factor Extraction method	Dataset	Factor Rotation Technique											
		No rotation			Varimax			Equamax			Quartimax		
		F1	F2	F3	F1	F2	F3	F1	F2	F3	F1	F2	F3
**Principal Component**	**Dataset 1** (40 Q-sorts): loaded Q-sorts dist. Statements changed statements^+^	22 5 -	6 7 -	2 10 -	13 8 5 (-2)	8 5 2 (-4)	10 3 2 (-4)^1^	10 7 2 (-5)^3^	10 2 1 (-6)	9 6 2 (-3)^2^	20 8 6 (-3)	8 6 4 (-5)	5 5 3 (-8)
	**Dataset 2** (33 Q-sorts): loaded Q-sorts dist. Statements changed statements^+^	29 8 -	1 15 -	3 7 -	17 5 1 (-4)	10 6 3 (-12)	5 8 3 (-2)	10 7 6 (-14)^2^	6 7 3 (-3)^3^	9 7 3 (-11)^2^	28 7 1 (-2)	2 9 4 (-2)^3^	1 16 3 (-2)^2^
**Principal Axis Factoring**	**Dataset 1** (40 Q-sorts): loaded Q-sorts dist. Statements changed statements^+^	21 5 -	6 9 -	1 8 -	11 8 6 (-3)	8 6 3 (-6)	8 4 3 (-7)	10 5 3 (-6)^3^	10 3 2 (-8)	7 5 2 (-6)^2^	19 6 4 (-3)	7 5 2 (-6)	4 5 2 (-5)

+ Number of changed statements includes both the new statements (positive number) and the statements that did not reappear in the new rotation (negative numbers) compared to *no-rotation*.

^1^ Matched with Factor 1 of no-rotation.

^2^ Matched with Factor 2 of no-rotation.

^3^ Matched with Factor 3 of no-rotation.

Additionally, in Dataset 1, the number of distinguishing statements for Factor 2 from no-rotation with PAF changed from 9 to 6, 3, and 5 for Varimax, Equamax, and Quartimax rotations, respectively. It is important to notice that although there might be some common statements in the distinguishing statements from different rotations, the factor scores can be, and usually are, quite different. Therefore, having some common distinguishing statements does not indicate sameness in the factors.

In addition to the number of loaded Q-sorts and number of distinguishing statements, Table 1 also shows that the number of distinguishing statements changed from *no-rotation* to other rotation techniques. For example, in Dataset 1, there were 7 changes in the number of distinguishing statements for Factor 1 based on Varimax rotation: 5 new statements that were not included in no-rotation, as well as 2 statements which were present in no-rotation but did not re-appear in Varimax rotation.

### Correlations between factor scores based on rotation technique

The other complexity which occurs due to different rotations is that as the number of participants and distinguishing statements change for each factor, the factor itself might become quite different from the previous rotation (or no-rotation). Table 2 includes the correlations between factor scores (z-scores) for different factor rotations based on PCA factor extraction for the two datasets, where the bold numbers indicate significant correlations. Next, each rotated factor from Varimax, Equamax, and Quartimax rotations was matched with a factor from no-rotation using the largest absolute value of correlation (marked with asterisk) (see next section). As shown in Table 2, one factor of no-rotation could be significantly correlated with more than one factor from another rotation. For instance, in both datasets, Factor 1 from no-rotation was significantly correlated with all 3 extracted factors from Varimax and Equamax rotations.

**Table 2:** Correlation matrix between factor scores based on principal component extraction and different rotation techniques.

**Dataset 1: Marijuana Legalization**										
		**No-rotation**			**Varimax**			**Equamax**		
		**F1**	**F2**	**F3**	**F1**	**F2**	**F3**	**F1**	**F2**	**F3**
**Varimax**	**F1**	**0.72** ^*^	0.03	**-0.69**						
	**F2**	**0.54**	**-0.64** ^*^	**0.54**						
	**F3**	**0.43**	**0.77** ^*^	**0.48**						
**Equamax**	**F1**	**0.64**	0.02	**-0.77** ^*^	**0.99**	-0.08	-0.08			
	**F2**	**0.56**	**0.67** ^*^	**0.49**	0.09	0.14	**0.99**			
	**F3**	**0.52**	**-0.74** ^*^	0.41	0.07	**0.99**	-0.15			
**Quartimax**	**F1**	**0.95** ^*^	-0.23	-0.23	**0.83**	**0.54**	0.12	**0.78**	0.27	**0.57**
	**F2**	0.32	**0.77** ^*^	**0.55**	-0.13	-0.02	**0.99**	-0.20	**0.96**	-0.17
	**F3**	-0.05	**0.60**	**-0.80** ^*^	**0.54**	**-0.84**	0.06	**0.60**	-0.02	**-0.80**
**Dataset 2: Childhood Obesity**										
		**No-rotation**			**Varimax**			**Equamax**		
		**F1**	**F2**	**F3**	**F1**	**F2**	**F3**	**F1**	**F2**	**F3**
**Varimax**	**F1**	**0.74** ^*^	**-0.65**	-0.16						
	**F2**	**0.54**	**0.72** ^*^	**-0.44**						
	**F3**	**0.40**	0.24	**0.89** ^*^						
**Equamax**	**F1**	**0.60**	**-0.75** ^*^	-0.26	**0.98**	-0.10	-0.17			
	**F2**	**0.58**	0.19	**0.79** ^*^	0.19	0.11	**0.98**			
	**F3**	**0.55**	**0.63** ^*^	**-0.55**	0.08	**0.99**	-0.12			
**Quartimax**	**F1**	**0.99** ^*^	-0.11	-0.10	**0.82**	**0.50**	0.27	**0.71**	**0.47**	**0.53**
	**F2**	0.13	0.28	**0.95** ^*^	-0.23	-0.14	**0.96**	**-0.38**	**0.88**	-0.28
	**F3**	0.08	**0.95** ^*^	-0.29	**-0.52**	**0.85**	0.00	**-0.60**	-0.01	**0.80**

* Matched factors with no-rotation factors.

Table 3 presents the correlation matrix between different factor rotations for Dataset 1 with PAF extraction. The results are very similar to Table 2. As mentioned before, an important finding is that the differences in correlation matrix based on different factor extractions are not as pronounced as the differences between different factor rotations.

**Table 3:** Correlation matrix between factor scores based on principal axis factoring extraction and different rotation techniques.

**Dataset 1: Marijuana Legalization**										
		**No-rotation**			**Varimax**			**Equamax**		
		**F1**	**F2**	**F3**	**F1**	**F2**	**F3**	**F1**	**F2**	**F3**
**Varimax**	**F1**	**0.75** ^*^	0.01	**-0.65**						
	**F2**	**0.54**	**-0.65** ^*^	**0.53**						
	**F3**	**0.46**	**0.75** ^*^	**0.48**						
**Equamax**	**F1**	**0.67**	0.01	**-0.74** ^*^	**0.99**	-0.04	-0.04			
	**F2**	**0.58**	**0.67** ^*^	**0.48**	0.13	0.12	**0.99**			
	**F3**	**0.55**	**-0.73** ^*^	0.42	0.13	**0.99**	-0.10			
**Quartimax**	**F1**	**0.96** ^*^	-0.21	-0.17	**0.84**	**0.57**	0.20	**0.77**	0.33	**0.61**
	**F2**	0.31	**0.78** ^*^	**0.56**	-0.13	-0.05	**0.98**	-0.19	**0.95**	-0.18
	**F3**	-0.04	**0.60**	**-0.80** ^*^	**0.51**	**-0.83**	0.05	**0.58**	0.01	**-0.79**

* Matched factors with no-rotation factors.

### Matching rotated factors with no-rotation factors

Given the changes in the factor scores from one rotation to another, we matched each rotated factor with a factor from no-rotation. For instance, for Dataset 2, Factor 1 from Varimax rotation was highly correlated with Factor 1 and Factor 2 of no-rotation, but it was matched with Factor 1 because it had the largest absolute correlation with this factor. Also, Factor 1 from Equamax rotation was highly correlated with Factor 1 and Factor 3 of no-rotation but it was matched with Factor 3 because it had the largest absolute value of correlation with this factor (i.e., -0.74).

### Changes in distinguishing statements

It might be suggested that the similarity between factors can be evaluated based on the similarity of the distinguishing statements. Table 4 presents distinguishing statements for Factor 1 of Dataset 1 with PCA factor extraction and its matched factors from Varimax, Equamax, and Quartimax rotations. Factor 1 had five distinguishing statements with no-rotation. The distinguishing statements for the matched factor with their factor scores are listed for the other rotations. Of the five distinguishing statements of no-rotation, only 3 re-appeared in any of Varimax and Equamax rotations, and only 2 in Quartimax rotation. The factor scores for the shared statements are also quite different. In addition, there are 5, 3, and 6 new statements in Varimax, Equamax, and Quartimax rotations, respectively, that were not identified as distinguishing statements in the no-rotation approach. Therefore, neither the shared statements with different factor scores nor different statements in the matched factors indicate sameness of the factors from no-rotation to any other rotation.

**Table 4:** Changes in distinguishing statements from no-rotation to the other rotation methods for Factor 1 from Dataset 1 with PCA factor extraction. Statements in bold show distinguishing statements from no-rotation that re-appeared in the other rotations.

Rotation	Statement	Factor 1	Factor 2	Factor 3
**No rotation**	8- Education and regulation are better options than prohibition 10- If we legalize marijuana, we reduce the black market and the violence associated with the sale of marijuana 3- The reason that marijuana poses a health threat is because most people smoke it, and smoking anything is hazardous to your health 14- By legalizing marijuana, more people will use the drug and as a result, become addicted and more families will become dysfunctional 12- The use of marijuana as a pain control may cause patients to rely solely on the drug rather than medical treatment	3 1 -1 -2 -3	0 -1 1 3 0	-1 -3 1 -1 2
**Varimax**	**10- If we legalize marijuana, we reduce the black market and the violence associated with the sale of marijuana** 4- Taxpayers are forced to pay billions of dollars to persecute, prosecute, and lock up people for having marijuana. If marijuana were legal, this money, plus tax revenues from marijuana sales, could be used for other purposes such as education or health care 19- If marijuana were legal, steps could be taken to reduce the health risks associated with its use by avoiding contamination 6- Marijuana legalization insures that people who use the drug for medicinal purposes, such as pain control, have access to it 2- By legalizing marijuana, doctors may become part of the black market by handing out prescriptions to those who want it rather than those who need it **14- By legalizing marijuana, more people will use the drug and as a result, become addicted and more families will become dysfunctional** **3- The reason that marijuana poses a health threat is because most people smoke it, and smoking anything is hazardous to your health** 13- Marijuana legalization would decrease the likelihood of younger children buying marijuana	2 1 1 0 -1 -1 -2 -2	0 0 0 2 -2 -3 1 0	0 -1 0 2 -3 1 1 -1
**Equamax**	**10- If we legalize marijuana, we reduce the black market and the violence associated with the sale of marijuana** 4- Taxpayers are forced to pay billions of dollars to persecute, prosecute, and lock up people for having marijuana. If marijuana were legal, this money, plus tax revenues from marijuana sales, could be used for other purposes such as education or health care 19- If marijuana were legal, steps could be taken to reduce the health risks associated with its use by avoiding contamination 6- Marijuana legalization insures that people who use the drug for medicinal purposes, such as pain control, have access to it **14- By legalizing marijuana, more people will use the drug and as a result, become addicted and more families will become dysfunctional** **3- The reason that marijuana poses a health threat is because most people smoke it, and smoking anything is hazardous to your health** 13- Marijuana legalization would decrease the likelihood of younger children buying marijuana	**3** **1** **1** **0** **-1** **-2** **-2**	**0** **-2** **1** **2** **0** **2** **-1**	**0** **0** **0** **1** **-3** **0** **0**
**Quartimax**	**8- Education and regulation are better options than prohibition** 17- Individuals should be allowed to choose whether or not they use marijuana; individual liberty is a fundamental value 18- There is an abundance of anecdotal evidence, as well as some scientific research, indicating that marijuana can be effective as a treatment for some illnesses 19- If marijuana were legal, steps could be taken to reduce the health risks associated with its use by avoiding contamination 11- It should become legal for those over the age of eighteen because these individuals are considered adults and are able to make their own decisions regarding drug use 6- Marijuana legalization insures that people who use the drug for medicinal purposes, such as pain control, have access to it 15- By legalizing marijuana, there will be an increase in people using the drug and therefore a need to increase rehabilitation programs which will come at the cost of taxpayers and the government **14- By legalizing marijuana, more people will use the drug and as a result, become addicted and more families will become dysfunctional**	**3** **2** **1** **1** **0** **0** **-1** **-3**	**1** **0** **2** **0** **-1** **3** **0** **1**	**1** **-3** **2** **0** **-1** **-1** **2** **3**

Therefore, comparing two factors only based on the quantitative criteria such as correlation between factor scores may not be appropriate and the potential interpretation of the factors can play a big role on answering the research question.

Although there are some high correlations between the factor scores from no-rotation with the matched factor scores from the other rotations (Tables 2 and 3), the interpretation of a factors is usually based on the distinguishing statements and statements with high scores. For example, compared with Factor 1 of no-rotation from Dataset 1 with PCA factor extraction, there are 2 fewer participants and 3 additional distinguishing statements for Factor 1 of Quartimax rotation (Table 1); yet, according to Table 2 the correlation between their factor scores is 0.95, which makes these two factors very similar quantitatively. However, there are only two shared distinguishing statements on these two factors (Table 4) which might make the interpretation quite different. Therefore, if there are substantial changes in the number and scores of the distinguishing statements it is highly likely that interpretations of the factor will change as well.

## Discussion

The effect of factor rotation is not well-known in Q-methodology. There have been some anecdotal discussions among Q-methodologists regarding the effects of factor rotation in the past. A recent book chapter claimed that factor rotation has little (if any) effect on the results of Q-methodology, specifically with relation to the distinguishing statements [9]. These discussions necessitate a thorough and practical investigation of such effects. However, such investigation might seem less necessary from a theoretical point of view because, historically, each new factor rotation was developed out of necessity to answer an important question.

Although the effects of factor rotation in Q-methodology have been examined in previous studies (for example see Akhtar-Danesh [1]), this article explores the impact of factor rotation more thoroughly. We specifically added the change in the number (Table 1) and scores (Table 4) of distinguishing statements for each rotation. We also investigated the changes in the correlations between extracted factors (Tables 2 and 3).

In the present study we showed that the effects of factor rotation on emerging factors is a complex and inter-twined process, and there is no one single criterion to define change in one factor. We examined several indices including the number of loaded Q-sorts on each factor, number of distinguishing statements for each factor, number of changed statements for each factor, Pearson correlation between factors scores from one rotation to another, and the changes in factor scores for each statement on each factor. This analysis showed substantial changes from one rotation to another such that the original no-rotation factor may not be recognizable in the rotated factors. For instance, there were only 3 distinguishing statements that remained unchanged between Factor 1 of no-rotation of Dataset 1 with PCA factor extraction and its matched factor from Varimax rotation. Even for these 3 common statements, the factor scores were quite different.

Finally, although we provided some quantitative criteria to assess the effects of factor rotation on the results of a Q-methodology analysis, the interpretation of a factor is usually based on distinguishing statements and statements with high scores. However, if there are substantial changes in the number and scores of the distinguishing statements of two factors, it is highly likely that interpretations of the factors will be different even if the factors are highly correlated.

In conclusion, factor rotation is a complex process that can affects several indices in Q-methodology. Among others, it can result in changes in the number and scores of distinguishing statements. These changes can be substantial such that the rotated factors might be quite different from the original factors. However, these changes may not affect all Q-studies equally and the differences may be negligible in some datasets or rotations. Although in this study we observed some substantial changes in the results based on factor rotation techniques, the magnitude of changes might be less or more pronounced on other datasets. Also, the changes in the findings from one rotation to another might be quite sensitive to the size of dataset, number of extracted factors, and rotation technique.

Currently, Varimax rotation is the most accepted approach for factor rotation in Q-methodology. However, the rotation method can change if the investigators have some hypothesis or prior knowledge of the dataset. For instance, if it is believed that there is a general factor in the dataset a Quartimax rotation may be more appropriate, or the investigator may use an oblique rotation if evidence suggests correlation between factors. As well, a theoretical (or manual) rotation can be used to examine whether specific participants load on a separate factor, though it would be difficult to justify such practice [19], specifically if participants are anonymous.

## Supporting information

S1 Appendix Key terms used in Q-methodology. (DOCX)

S2 Appendix List of statements for Dataset 1 (Marijuana Legalization). (DOCX)

S1 File Marijuana legalization dataset. (XLSX)

S2 File Childhood obesity dataset. (XLSX)

S3 File Statistical codes. (TXT)

## References

[1] Akhtar-Danesh N. A Comparison between Major Factor Extraction and Factor Rotation Techniques in Q-Methodology. Open Journal of Applied Sciences. 2017;7(4):147–56.

[2] Ramlo S. The Preferences of Q Methodologists at the Factor-Analytic Stage: An Examination of Practice. Researh in the Schools. 2017;24(2):40–5.

[3] Sneegas G, Beckner S, Brannstrom C, Jepson W, Lee K, Seghezzo L. Using Q-methodology in environmental sustainability research: A bibliometric analysis and systematic review. Ecological Economics. 2021;180:106864. 10.1016/j.ecolecon.2020.106864.

[4] Dieteren CM, Patty NJS, Reckers-Droog VT, van Exel J. Methodological choices in applications of Q methodology: A systematic literature review. Soc Sci Humanit Open. 2023;7:100404. 10.1016/j.ssaho.2023.100404.

[5] Schmolck P. PQMethod (Version 2.35, adapted from mainframe-program Qmethod written by John Atkinson, 1992)[Computer Software]. Retrived from http://schmolck.org/qmethod/. Neubiberg: University of the Bundeswehr Munich; 2014.

[6] Banasick S. KADE: A desktop application for Q methodology. Journal of Open Source Software. 2019;4(36):1360. 10.21105/joss.01360.

[7] Akhtar-Danesh N. qfactor: A command for Q-methodology analysis. The Stata Journal. 2018;18(2):432–46.

[8] Zabala R, Held M, Hermans F. Package ’qmethod’ (The package reference manual) 2023 [updated 28/5/2023]. Available from: https://cran.r-project.org/web/packages/qmethod/qmethod.pdf.

[9] Braswell RD. Comparative rotations and analyses of Q data: A worked example. In: Rhoads JC, Thomas DB, Ramlo SE, editors. Cultivating Q Methodology: Essays Honoring Steven R Brown. United States: International Society for the Scientific Study of Subjectivity; 2022. p. 145–81.

[10] Stephenson W. Technique of factor analysis. Nature. 1935;136:297.

[11] Stephenson W. Correlating persons instead of tests. Character and Personality. 1935;4:17–24.

[12] Akhtar-Danesh N, Baumann A, Cordingley L. Q-methodology in nursing research: A promising method for the study of subjectivity. Western Journal of Nursing Research. 2008;30(6):759–73. doi: 10.1177/0193945907312979 18337548

[13] Brown SR. A primer on Q methodology. Operant Subjectivity. 1993;16:91–138.

[14] McKeown B, Thomas DB. Q Methodology. 2 ed. Thousand Oaks, CA: Sage Publication; 2013.

[15] Watts S, Stenner P. Doing Q Methodological Research: Theory, Method and Interpretation. Los Angeles: SAGE Publications Ltd; 2012.

[16] Tabachnick BG, Fidell LS. Using Multivariate Statistics. 6th ed. Boston: Pearson; 2012.

[17] Akhtar-Danesh N. An overview of the statistical techniques in Q-methodology: Is there a better way of doing Q-analysis? Operant Subjectivity. 2017;38(3/4):29–36.

[18] Thurstone LL. Multiple Factor Analysis. Chicago, IL.: University of Chicago; 1947 1947.

[19] Akhtar-Danesh N, Mirza N. Relation between manual rotation and abductive reasoning in Q-methodology. Open Journal of Social Sciences. 2017;5:198–204.

[20] Gorsuch RL. Factor Analysis. 2nd ed. Hillsdale, NJ: Lawrence Erlbaum; 1983 1983.

[21] Kline P. An easy guide to factor analysis. New York, NY: Routlede; 1994 1994.

[22] Hair JF, Anderson RE, Tatham RL, Black WC. Multivariate data analysis with readings. 4th ed. Englewowd Cliffs, NJ: Prentice Hall; 1995 1995.

[23] Stephenson W. Scientific creed—1961: Abductory principles. The Psychological Record. 1961;11:9–17.

[24] Akhtar-Danesh N, Wingreen SC. qpair: A command for analyzing paired Q-sorts in Q-methodology. The Stata Journal. 2022;22(4):884–907. doi: 10.1177/1536867X221141002

[25] Akhtar-Danesh N, Dehghan M, Morrison KM, Fonseka S. Parents’ perceptions and attitudes on childhood obesity: a Q-methodology study. J Am Acad Nurse Pract. 2011;23(2):67–75. doi: 10.1111/j.1745-7599.2010.00584.x 21281372

[26] Holzinger KJ. A comparison of the principal axis and centroid factors. Journal of Educational Psychology. 1946;36(8):449–72. doi: 10.1037/h0056539 20283205

[27] Mulaik SA. Methods of Factor Extraction. Foundations of Factor Analysis, Second Edition. Chapman & Hall/CRC Statistics in the Social and Behavioral Sciences: Chapman and Hall/CRC; 2009. p. 139–65.

[28] Akhtar-Danesh N. Using Cohen’s Effect Size to Identify Distinguishing Statements in Q-methodology. Open Journal of Applied Sciences. 2018;8(2):73–9.

