# Supplementary material for: Impact of factor rotation on Q-methodology analysis
Source: PLoS One. 2023 Sep 1;18(9):e0290728. doi: 10.1371/journal.pone.0290728 (PMC10473483; doi:10.1371/journal.pone.0290728)
Supplement: S1 Appendix — (DOCX) [file pone.0290728.s001.docx]

**S1 Appendix.** Key terms used in Q-methodology

| Concourse | The initial set of statements collected from participant surveys, interviews and focus groups, review of prior concourses, literature review, etc. |
| --- | --- |
| Q-sample (Q-set) | A revised and representative list of statements from the concourse used by the participants for data collection. |
| Q-sort table | The table provided to participants for sorting statements within the Q-sample – this table has a quasi-normal distribution and should have as many cells as the number of statements in the Q-sample |
| Q-sort | A completed Q-sort table which includes a participant’s rank-ordered set of statements |
| Factor | A group of participants with similar viewpoints presented by the participants’ Q-sorts. Factors are extracted using a by-person factor analysis on all Q-sorts |
| Factor loadings | Factor loadings represent the correlations between participants and factors |
| Factor score | Denotes level of agreement between statement and a factor |
| Distinguishing statement | A distinguishing statement for each factor is a statement that its factor score for the factor is significantly different from its factor scores on all the other factors |
| Consensus statements | A statement that all participants (loaded on all factors) agree or disagree with at the same level. For a consensus statement, there is no statistically significant difference between factor scores across factors |
