# Supplementary material for: Impact of factor rotation on Q-methodology analysis
Source: PLoS One. 2023 Sep 1;18(9):e0290728. doi: 10.1371/journal.pone.0290728 (PMC10473483; doi:10.1371/journal.pone.0290728)
Supplement: S2 Appendix — (DOCX) [file pone.0290728.s002.docx]

**S2 Appendix**. List of statements for Dataset 1 (Marijuana Legalization)

| 1- The harms associated with marijuana are less than those associated with tobacco and alcohol, and they are not sufficient reason to justify making marijuana illegal.  2- By legalizing marijuana, doctors may become part of the black market by handing out prescriptions to those who want it rather than those who need it.  3- The reason that marijuana poses a health threat is because most people smoke it, and smoking anything is hazardous to your health.  4- Taxpayers are forced to pay billions of dollars to persecute, prosecute, and lock up people for having marijuana. If marijuana were legal, this money, plus tax revenues from marijuana sales, could be used for other purposes such as education or health care.  5- Marijuana does not cause violence. In fact, people who are high on marijuana tend to be relaxed, mellow, and too happy to want to fight.  6- Marijuana legalization insures that people who use the drug for medicinal purposes, such as pain control, have access to it.  7- Certain people are able to function only with the aid of marijuana. If it is more difficult for them to access the drug, they may be unable to perform effectively in society.  8- Education and regulation are better options than prohibition.  9- Prohibition is not an effective solution to the problems associated with marijuana use.  10- If we legalize marijuana, we reduce the black market and the violence associated with the sale of marijuana.  11- It should become legal for those over the age of eighteen because these individuals are considered adults and are able to make their own decisions regarding drug use.  12- The use of marijuana as a pain control may cause patients to rely solely on the drug rather than medical treatment.  13- Marijuana legalization would decrease the likelihood of younger children buying marijuana.  14- By legalizing marijuana, more people will use the drug and as a result, become addicted and more families will become dysfunctional.  15- By legalizing marijuana, there will be an increase in people using the drug and therefore a need to increase rehabilitation programs which will come at the cost of taxpayers and the government.  16- A decrease in the use of marijuana means fewer health risks such as slowed brain function or risk of cancer.  17- Individuals should be allowed to choose whether or not they use marijuana; individual liberty is a fundamental value.  18- There is an abundance of anecdotal evidence, as well as some scientific research, indicating that marijuana can be effective as a treatment for some illnesses.  19- If marijuana were legal, steps could be taken to reduce the health risks associated with its use by avoiding contamination. |
| --- |
